# Supplementary material for: The effect of game-based education on adherence to treatment and anxiety level in type 2 diabetics started on insulin therapy
Source: PLoS One. 2026 Mar 30;21(3):e0345292. doi: 10.1371/journal.pone.0345292 (PMC13035163; doi:10.1371/journal.pone.0345292)
Supplement: S1 File — (DOCX) [file pone.0345292.s001.docx]

*
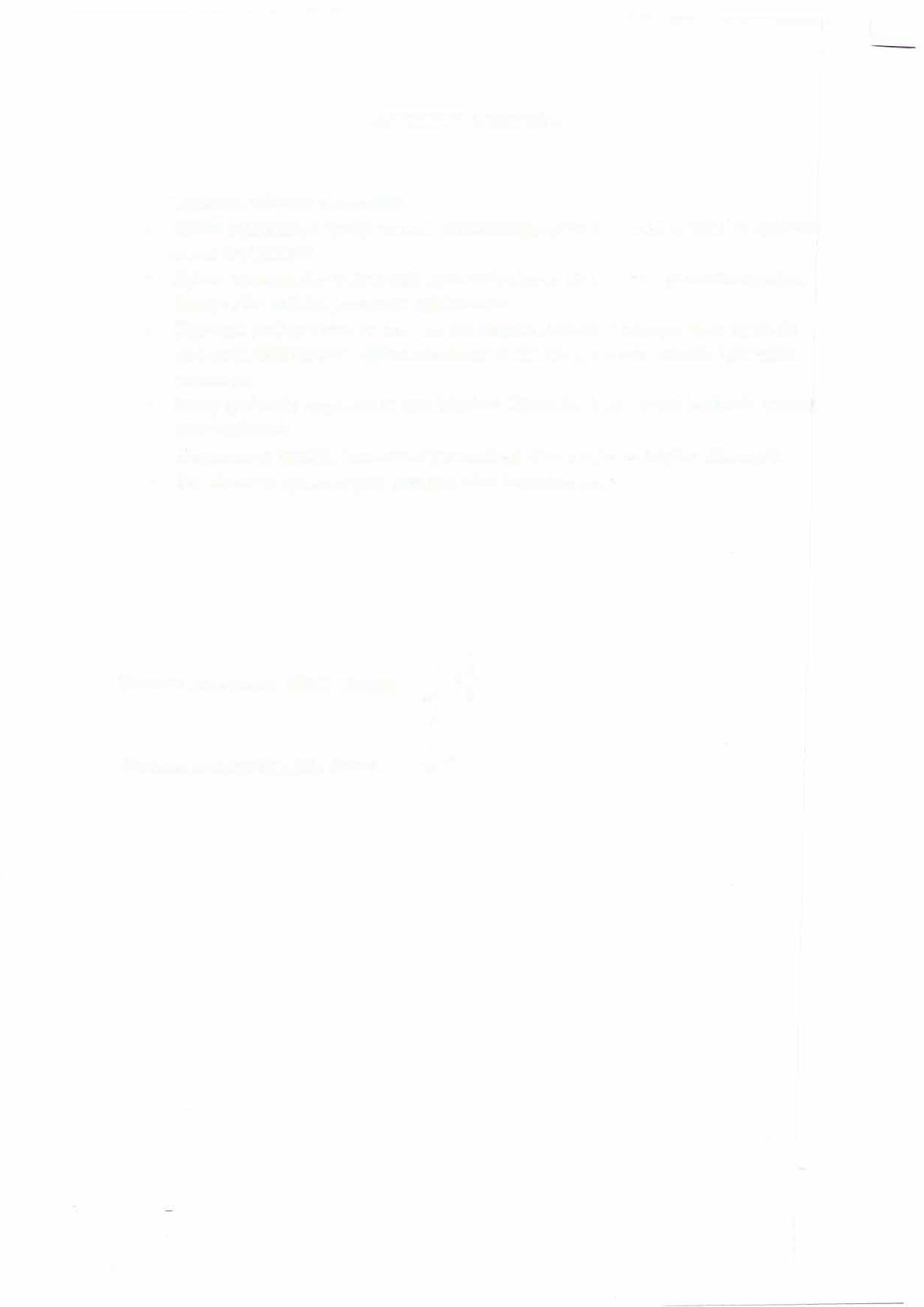
REVISION LETTER*

- *The exclusion criteria has been added.*
- *A detailed table of content of the training program and how to apply and is explained in the description.*
- *Training sessions, according to the game level in the experimental group, the subjects in the control group, according to the weekly program, are described.*
- *A total of seven interviews pre - and post-test assessment (two interviews at the beginning and end), five sessions of training in the description for the experimental and control groups were reviewed.*
- *The table out of the game, which will be applied in the experimental group are given detailed information about diabetes.*
- *In the scope section of research under other item info added has been deleted.*
- *Thesis work in process-time is described with the schedule table.*

# Principal Investigator:

# Assistant Researcher:

LEARN THE GAME OF DIABETES

(EXPERIMENTALTRAINING GROUP TO BE APPLIED IN GAME- BASED DESIGN) DURATION: 45-60 MINUTES

Objective: the aim of the training process still to come motivating obtainayan methodsin education has been the introduction of one of oyunlastirman.Educationteam dramatization of the individual in the educational process with the goal is to get more active andtrainidid stres to being a donor state. With this method while having fun individual thatGlearning, and aims to increase efficiency in education. To learn new information, a strong fiphysical, cognitive effort, and self-regulation requires. Individuals with diabetes the aim of the gamer, teachers,students, playing games, and playsall for people with diabetes farkindalik oon a whole. The basic information that will make life easier for individuals with diabetes, with diabetes asthe ERin ioyunlastirilm ensure that nteraktif format. Also in the school of diabetes diyabet given by individuals with diabetes education nurses into the game motivaSchool of healthyou provide.

MATERIALS that can be used on floor or table bz ir mainsure the carpet of the game, DICE, card question and answer cards for each level for a total of 69 23 (birinci, easy level, second-level middle level hard-to questions and issues in the category ofr -ir), flipchart blackboard

DESCRIPTION: according to the number of players participating in two or uc tCurrentis divided into a. A spokesperson from the team is selected, and the dice is thrown. Will start the game, the dice teampickielicitation fromthen game on the carpet, 1from 23tonumbers, respectively, for each team inthe middleof deciding to choose one that makes a card number and K is written. Card inalsoruy nowu answering correctly, and the team gets a point guidelines. The figures in order actirilIR and until the end of the teams by the facilitator, the scores are recorded.

GAINS:

- Describes basic information about diabetes
- Emergencies and describes interventions in diabetes
- The correct steps for the measurement of blood glucose at home,aCIto Dandapply
- Individuals with diabetes have blood glucose values aciklar.
- Explains and demonstrates the application of the steps of insulin.
- Understand and explain the importance of exercise in diabetes.
- Diabetes is caused by long-term health problems and theseverityknows download
- in Diabetes Foot Care, explains and applies.

Principal Investigator:

Assistant Researcher:

BUTLER UNIVERSITY

NON-INTERVENTIONAL CLINICAL RESEARCHLAR ETHICS COMMITTEE

**ThesisSul Work-Time Schedule Plan**

| work**PackageNo** | Jobpaket name definition |  | | | |  | | | |
| --- | --- | --- | --- | --- | --- | --- | --- | --- | --- |
|  |  | **Ohammer-March**2024 | February-  **l\,l;IRT**  **2024** | May - July**2024** | July  2024 | , Agus.-semp.  2024 | October | December  2025 | January- February  ·2025 |
| l | Lltentur Scan | ✓ |  |  |  |  |  |  |  |
| 2 | scan sourcesve notAlina |  |  | ✓ |  |  |  |  |  |
| 3 | thesis subject of theflourclarity.develop | ✓ |  |  |  |  |  |  |  |
| 4 | the universe is determinedleme andthatmjoint selection |  |  | ✓ |  |  |  |  |  |
| *5* | Ethicalk:iirul to take permission of the institution |  | ✓ |  |  |  |  |  |  |
| *6* | Kontrol groups pre-test and the implementation of the scales |  |  |  | ✓ |  |  |  |  |
| 7 | experiments and kontroll groupsbes otype Ideducation U ofyof gulanma,son scale implementation of Test |  |  |  |  | ✓ |  |  |  |
| 8 | data entry andanalysis |  |  |  |  |  | ✓ |  |  |
| *9* | writing the thesis |  |  |  |  |  |  | ✓ |  |
| *9* | of thesisIZTUgraduate educationEnstitusune the delivery |  |  |  |  |  |  |  | ✓ |

Figure:2

10

BUTLER UNIVERSITYI

NON-INTERVENTIONAL CLINICAL RESEARCHIRMAETEAK BOARD

**DESCRIPTION: by the number of players participating in twoor even three teams are divided into two. A spokesperson from the team is selected, and the dice is thrown. Start the gameguide t- current membrane is selected with the game after you on the carpet, and from 23 to 1, Rak- amlarorn, respectively, where K is written, giving each team a joint decision and choose one of that numberartlari actirir. The team with the correct instruction in the card answering your question and pumoment ka -zaNir. The figures and the scores of the teams in order until the end actirili facilitator tarafSdan are recorded.**

**GAINS:**

- **Diabetes**about**describes basic information**
- **Emergencies and describes interventions in diabetes**
- **The correct steps for the measurement of blood glucose at homev describesand implements e**
- **Individuals with diabetes have blood glucose valuesaCIfor.**
- **Explains and demonstrates the application of the steps of insulin.**
- **Understand and explain the importance of exercise in diabetes.**
- **Diabetes is caused by long-term health problemlArininthatmoisture. ini knows**
- **Foot care in diabetes, explains and applies.**

The data obtained in the study, SPSS (statisticalPackage for Social Sciences)for Windows

25.0 will be analyzed using a program. The data alsogerlendirilidefine whileyICI statistical methods (number, percentage,min-max values, ortalbut and standard deviation) will be used. In order to test the reliability of the scalesyla "reliability analysis" will continue to apply.

9

**BUTLER UNIVERSITY**


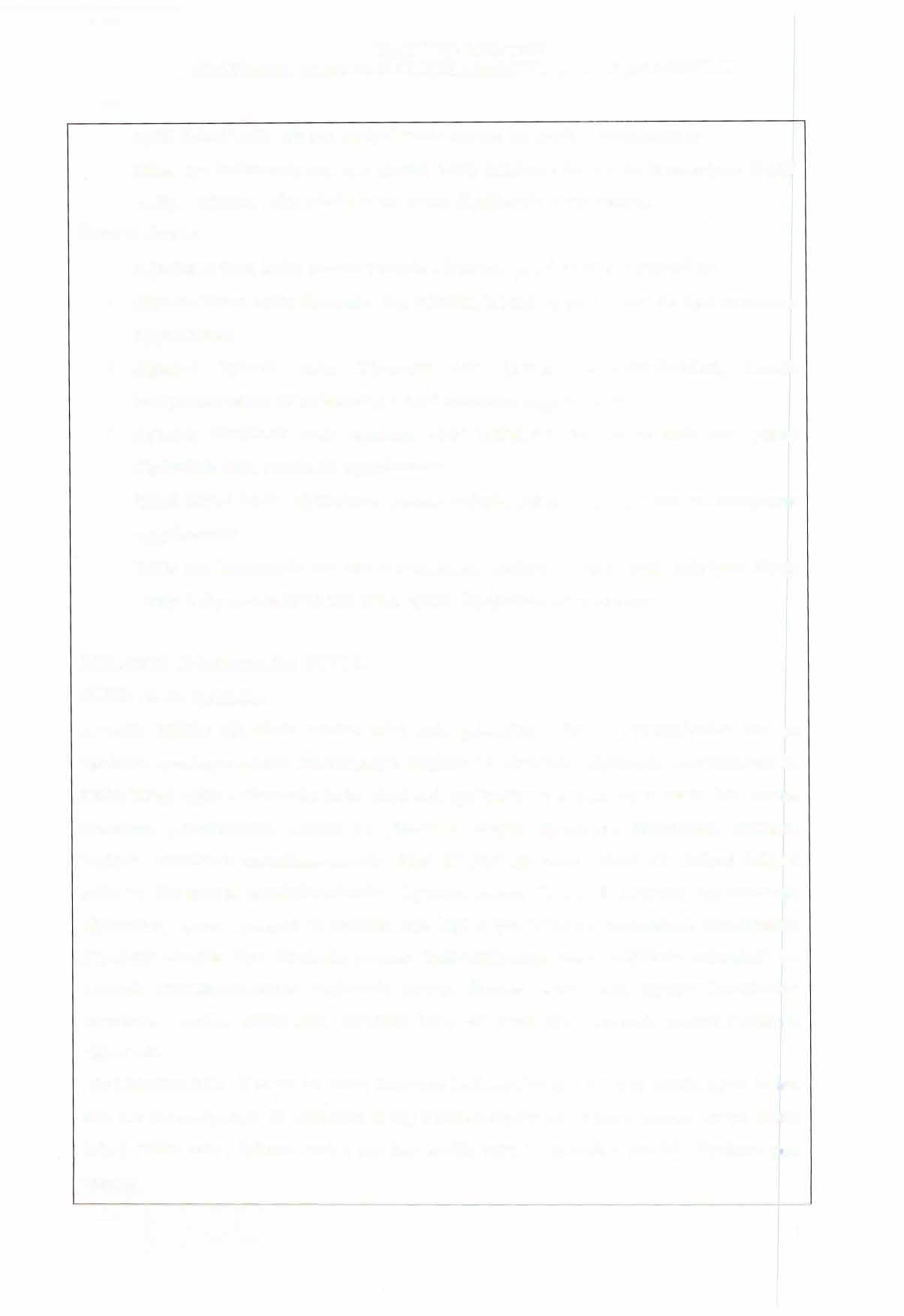


**NON-INTERVENTIONAL CLINICAL RESEARCH ETHICS BOARD**

- **The second week of September of the game's levels is applied in a mixed manner**
- **During the last week of October as a final test, the patient diagnosis form,beck anxiety scale and patient compliance in the treatment of Type 2 diabetes olcegin of implementation**

**Control Group**

- **The first week of August will be given through the presentation of general information on diabetes**
- **The second week of August, diabetes medication, insulin about the practices of implementation of the presentation**
- **August the third week in diabetes emergency situation and interventions, and measures on implementation of the Chronic Complications of the presentation**
- **The fourth week of August, exercise, medical nutritiontedavisia presentation on the implementation and measurement of blood glucose at home**
- **Training the second week of September through the presentation of a general review of the implementation questions and answers**
- **During the last week of October as a final test, the patient diagnosis form,beck anxiety scale**

**patient compliance in the treatment of Type 2 diabetes andolcegin of implementation**

**OUT OF THE GAME, DIABETES DURATION: 45-60 MINUTES**

**OBJECTIVE: one of the ways of motivating training process that enables us to still come in education has been the introduction of oyunlastirman. Dramatization of the individual in the educational process Education and training with the goal to make a stressful situation is subtracting it from being more active. Also with this method the individualeglenirk,en learning, aims to increase efficiency in education. To learn new information, a strong physical, cognitive effort, and self-regulation requires. The aim of the game individuals with diabetes, teachers, students, playing games, and plays in diabetes is to create awareness for all people. For individuals with diabetes with diabetes oyunlastirilm ensure that the basic information in an interactive format that will make life easier. Diabetes mellitus is also given by nurses in schools of education into the game by adding motivation to provide individuals with diabetes.**

**MATERIALS that can be used on floor or tablebir main gaming floor carpet, dice, card, cards for a total of 69 23 question and answer for each level (the first level is easy, the second level of the middle third category includes topics and questions in the difficult level),flipchart whiteboard**

.

**Non-interventional CLINICAL ARASTIRMALARETHR BOARD**

has a positive effect on.

H0: individuals with Type 2 diabetes given game-based training, one-on -ylerinaPTsihas a positive effect on the level of em.

Hl: individuals with Type 2 diabetes given game-based training,bireysn has a positive effect on the level of anxiety

Butler training and Research Hospital, Internal Medicine, Endocrine and polyklflat/kldown to the last by contacting

insulin therapy was initiated three monthsve Diabetes Education Unitnehead inpatients with Type 2 diabetes who hit for

**Inclusion Criteria**

- Age at diagnosis of diabetes have at least one year
- Insulin therapy is now more than three months time baslanma
- Mental problems, lack of
- 40 - 65 years be in the range of
- Willingness to participate in the research criteria such as kRSilMoonanpatients will be included in the research.

**Exclusion Criteria**

- - **New age of diagnosis of diabetes, or less than a yearolmaSI**
  - **For more than three years taking insulin therapyin MASI**
  - **Mental or psychological disease to be additionalsI**
  - **The target age range in the absence under the age of 40or 65 orSonrinbe**
  - **Accepting to participate in the research**
  - **In the research process consists of five sessions, trainingsessionswith one or more of Rin I couldn agree**

**Data collection tools andMethods**- **that will be used for statisticalManagementemler**

Pre - and post-test randomized control grupiu Experimental ShrubSMA IIzdscales to be used in a form and the form of the voluntary consent of the Patient Assessment Form, Beck Anksiyete Scale, Type 2 Diabetes Mellitus is the scale of patient compliance to treatment.

**NON-INTERVENTIONAL CLINICAL RESEARCHSETIK BOARD**

elements increases the motivation of the person (4,5). YapIladramatization of applications in the literature for protection from diabetes healthy n beslenme, physical activity programs that can be developed and implemented in conjunction with ifadE Edilebilyre. Nutrition plans for patients, the blood glucose measurements and insulin use miktarlRSS K amonthwhere dedild my oyunlastii the motivation of individuals in the fight against diabetes apps artIrarak can increase the effectiveness of treatment, can facilitate patient tracking, and unnecessary hospital ziyaretlerini can reduce. This, can contribute to the effectiveness and efficiency of Health Services. Burada damong the issues to be aution, destination, entertainment, and will include the appropriate tools sekilde dramatization of the design process and the establishment of an accurate location. However, diyadramatization bet's disease in the context of the application to the height of the input frequency nedeniylekatrialHR non-right of your attitude and behavior is rewarded applications where geCIRrecognizing the Varietyis expressed that this will increase the effectiveness of oyunlastirma (6). Game-based Training Mis very often used in different areas of business models in the field of education, while very limited in the field of Health countIDAis. However, a group of diseases such as diabetes that are difficult to manage anxiety and harmony in the jungle ofyi , so thatneis Dubrovnik. All diabetics, especially age, physical disability, complications due technolojiHe judge might not. Games to learn by having fun because it is their right her yaStmoment, from every culture designed to accommodate individuals with diabetes, a simple and straightforward structure. NowcECtable out of digital media or board games has not been found. For these reasons arastirmacI and colleagues designed by diabetes-related games with Type 2 diabetes bireylerin tedavharmonies UTI, anxiety levels are randomized in order to investigate the effects of kontrollu an experimental study will be conducted.Game-based learning models with Type 2 diabetes, bireyin a positive impact on treatment compliance and reduce the level of anxiety is subject to thesunda is considered to contribute to further.

**RESEARCH MATERIAL AND METHOD**

**Hypotheses:**

H0: individuals with Type 2 diabetes given game-based training,bireylerin compliance has a positive effect on diabetes treatment.

Hl: individuals with Type 2 diabetes given game-based learning,bireylerincompliance to diabetes treatment

5

**BUTLER UNIVERSITY**


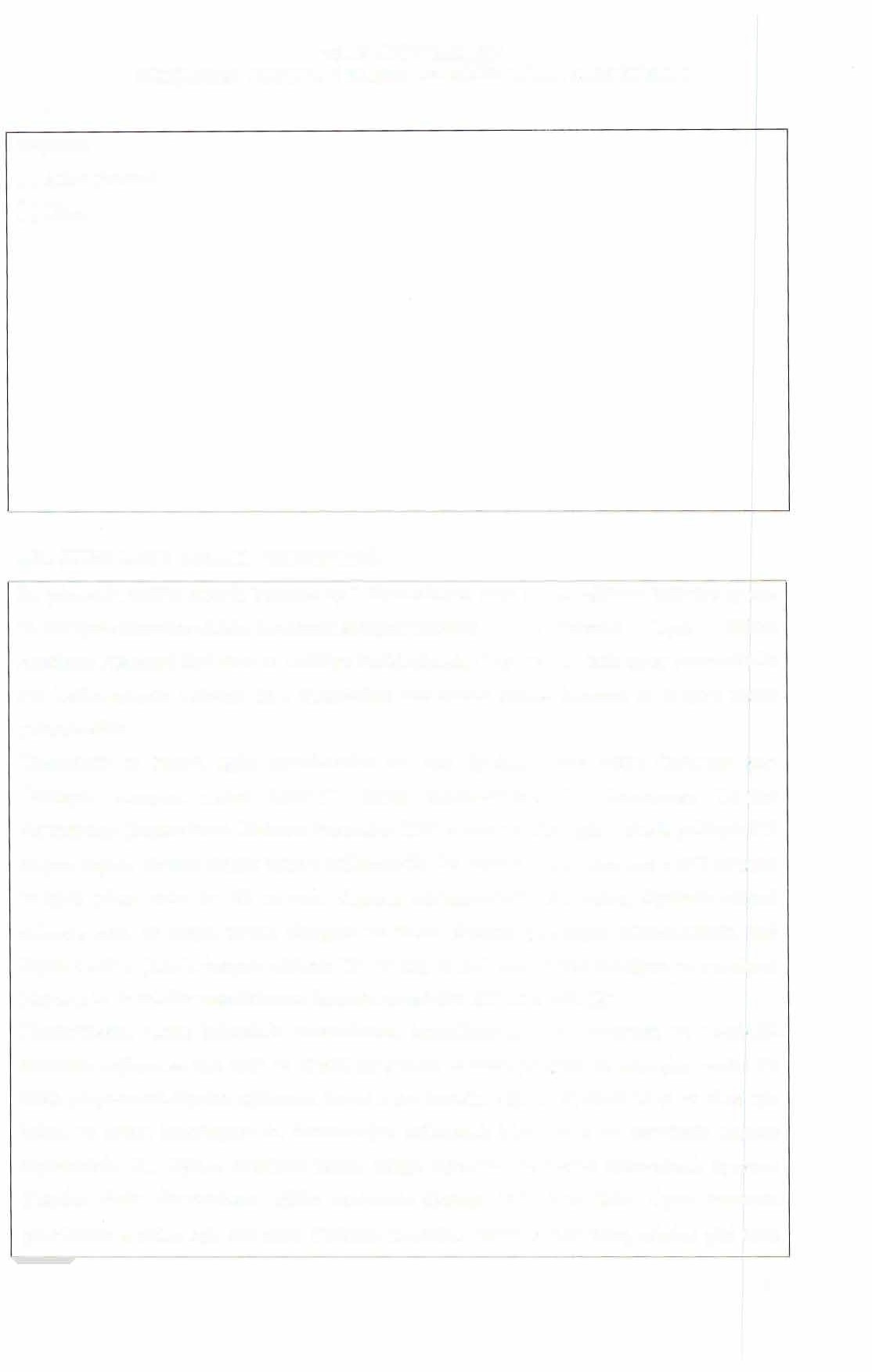


**NON-INTERVENTIONAL CLINICAL RESEARCH ETHICS BOARD**

Research

O Survey method

O Other:

# STUDY PURPOSE/ RATIONALE:

In this study, Type 2 diabetes insulin treatment was started in oyw1-based training for the treatment of sleep and examine the impact on the level of anxietyit is intended to . Butler arastim1amiz training and Research Hospital, Internal Medicine, Endocrine and outpatient clinics for the first time in more than three months, the applicant including Type 2 diabetes insulin treatment was started as a wide range of experimental study will be conducted
Today's most important health sornn of which is diabetes, World Health Organization , "according to non-communicable epidemic has been described as" (1 ). The International Diabetes Federation (Intemational Diabetes Federation,IDF), according to 537 million people are estimated to have diabetes in approximately 2021. 783 643 million and by 2030 this number is expected to reach million until the year 2045. Diabetes is an increasing health these data in a global sensesornnu and suggests that precautions should be taken. IDF Diabetes Atlas (2021), the adult population (20-79 years), and 10.5% of those living with this disease, and diabetes are not aware of reveals that almost half (2)

Improving the quality of life of diabetes complications and metabolic control for the provision of effective protection and are in need of regular maintenance and monitoring program. The importance of this monitoring program, diabetes education is inevitable. Education for individuals with diabetes and their families to facilitate the care and monitoring, to ensure continuity knowledge, attitudes and skills must include all of (3). Educational gamesjudge ortan1larina learning game-based learning environments is called. Game-based learning is active learning model. There are possibilities for receiving feedback for correcting errors during the game. Also the game as a racing competition

4


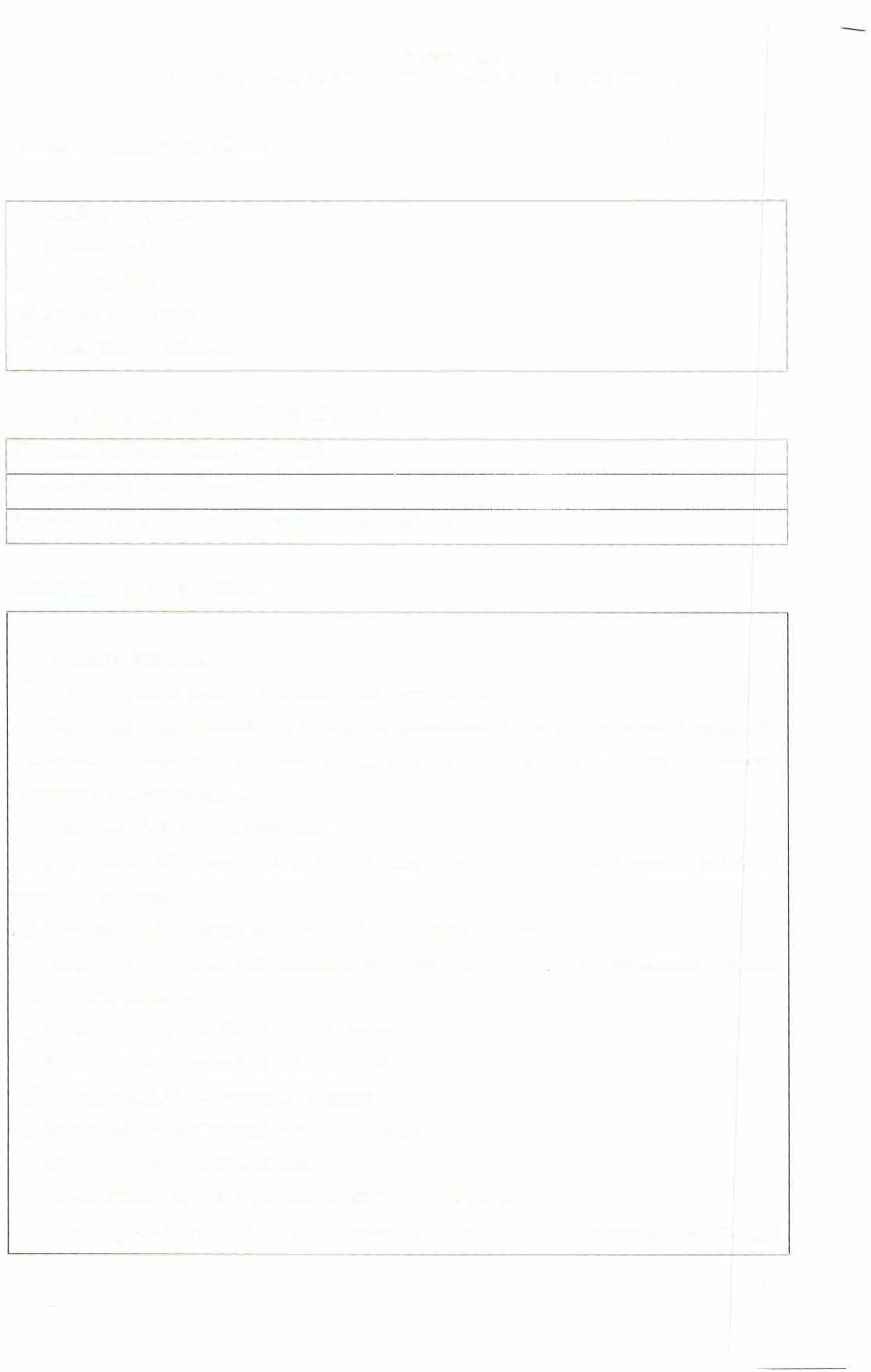


**NON-INTERVENTIONAL CLINICAL RESEARCH ETHICS BOARD**

# THE NATURE OF THE RESEARCH

Individual Research

PhD Thesis

PhD.

Thesis

Other (specify type):

# THE PROJECTED DURATION OF THE STUDY:

Planned Start Date: May 2024Planned End Date: February 2025

Budget and Source: and there is no support budget.

**THE SCOPE OF THE RESEARCH**

observational research

retrospective scan and image such as records archive File

blood, urine, tissue, such as images, Biochemistry, Microbiology, patoloji collection and radiology

materials or routine inspection, examination, diagnosis and cure obtained during the processwith mateiyal research

cell or tissue culture research

gene therapy outside of clinical research, and for defining geneticmateiyal with research

Nursing activities within the boundaries of research to be done

computing environment for testing, interviews, data to be collected by the research use audio or video recording of

Biomedical and/or research on ecology

health education activities related to research

anthropometric measurements based on research

research on the physiology of the body, such as Exercise

Nutrition/diet-related research

Life habits related to the evaluation of researches

perfom1ans assessment related to in vitro diagnostic medical devices are made with

3

**BUTLER UNIVERSITY**

**NON-INTERVENTIONAL CLINICAL RESEARCHS ETEAK BOARD**

| Other researchers: | | | | | |
| --- | --- | --- | --- | --- | --- |
| name and surname of inspiration: | | | | | |
| Title: Nurse, | | | | | |
| Doctor/Specialty/other interests: health care communication in | | | | | |
| business address: | | | | | |
| phone number: | | | | | |
| e-mail: | | | | | |
| the responsibility of research/Contribution: | | | | | |
|  | □ | Hypothesis Development | □ | ResearchYiplmomentlama |  |
|  | □ | data collection | □ | main Dataliziandyorumlama |  |
|  | □ | article writing |  | Other: YuL highisans Thesis |  |
| Signature: | | | | | |

I,

I,

2

**NON-INTERVENTIONAL CLINICAL RESEARCHSETEAK BOARD**

**Application Deadline**

Revision Date

**Full name of the research:**Insulin treatment was started, type 2 diabetes, lilpremature ineffect on the adherence to the treatment of anxiety level and game based learning

| **Principal investigator** | | | | | | |
| --- | --- | --- | --- | --- | --- | --- |
| name, surname: | | | | | | |
| Title: Dr. faculty member, | | | | | | |
| Doctoral/research interests: : | | | | | | |
| business address: | | | | | | |
| telephone number: | | | | | | |
| e-mail: | | | | | | |
| responsibility in research/Contribution: | | | | | | |
|  | □ | hypothesis development | | □ | ArastinnayI planned, but |  |
|  | □ | data collection | | □ | data analysis, andy -orumlama |  |
|  | □ | essay writing | | supporting: | Other:Load -figLiTuin NS advisor |  |
|  | | | | | | |
| Signature: | | |  | | | |
|  | | | | | | |

**BUTLER UNIVERSITY**


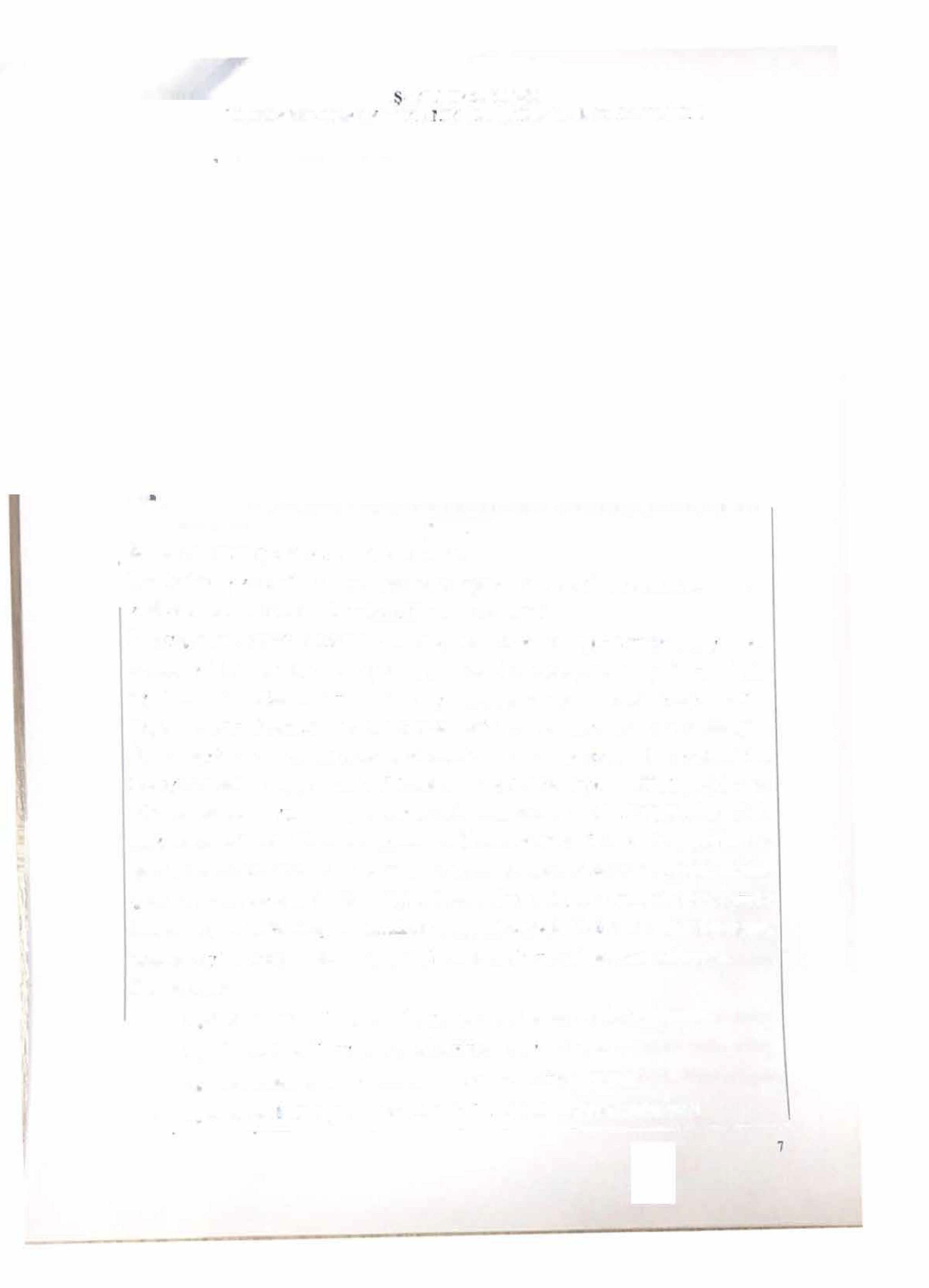

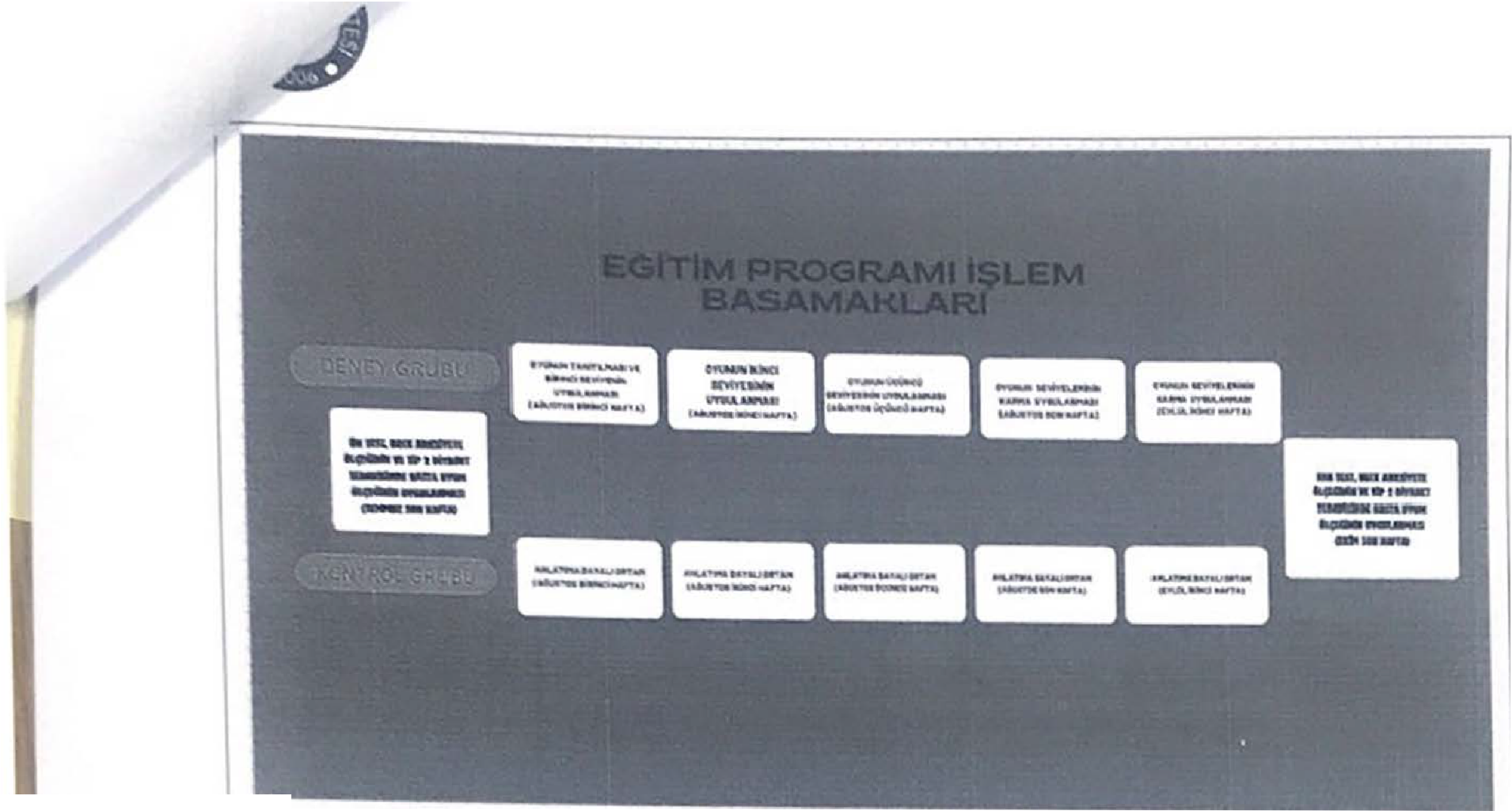

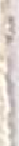


**NON-INTERVENTIONAL CLINICAL RESEARCH ETHICS BOARD**

**Figure**- **1training program content and process steps**

**\The patientsin the experimentaland control group will be divided into two groups, all pre-Test form**

**the study and the scales will be pre-filled (last week of July).**

**Try the group out of the table designed by the researcher to diabetes 0)flour four sessions in the first month, in the second month, with a total of five training session, the session will be applied. At the same time the control group presentation (lecture) will be applied to education through (Figure: 1). The average duration of the session is planned as two hours. In the third month of the last Test will be conducted as the form and scale of the latest reviews. Try)training and Research Hospital Diabetes Education and control groups will be implemented in the school hall for this Butler.Diabetes school gym for 16 persons in totalpower analysis the sample size was 64 (32 in the experimental group, 32 control group) for the experimental group to be seen and in front of each session in the morning and in the afternoon (around 16 to 16), for the control group in the morning \'e in the afternoon (around 16 to 16) divided into two groups experimental group control group on a different day on a different day so it will be the first** month for **one day per week, for the second month)one day each group will participate in a total of five training sessions.**

**The Experimental Group**

1

- **Introduction and implementation of the first level of the game for the first week of August**
- **implementation of the second level of the game in the second week of August**

*I*

- **The third level of implementation of the game of the third week of August**
- **The game's levels last week of August is applied in a mixed manner**

'--
