# Supplementary material for: The effect of game-based education on adherence to treatment and anxiety level in type 2 diabetics started on insulin therapy
Source: PLoS One. 2026 Mar 30;21(3):e0345292. doi: 10.1371/journal.pone.0345292 (PMC13035163; doi:10.1371/journal.pone.0345292)
Supplement: S2 File — (DOCX) [file pone.0345292.s002.docx]

**Description of the Game-Based Educational Intervention**

The *“Let’s Learn Diabetes”* board game is an educational tool developed by the researchers based on a game-based learning model and registered with the Turkish Patent Office (Design Registration No: 2023 013290). The game was designed to support diabetes education through active participation, peer interaction, and structured feedback, and was used as the primary educational intervention in this study.

**Game Structure and Materials**

The game consists of the following components:

- A large floor or table-based game board (game rug),
- Dice,
- A total of 69 question-and-answer cards divided into three levels of difficulty (easy, moderate, difficult; 23 cards per level),
- Flipchart boards for score recording.

Participants were divided into two or three teams. Each team selected a spokesperson, and teams took turns rolling the dice and moving on the game board. Based on the selected number, teams answered questions corresponding to the relevant difficulty level. Correct responses allowed teams to gain points and progress in the game. A trained facilitator (nurse educator) guided the sessions and recorded scores.

Each game session lasted approximately 45–60 minutes and was conducted as part of a structured educational workshop.

**Educational Content**

The content of the board game comprehensively covered the topics included in standard diabetes education programs, including:

- Basic concepts of diabetes mellitus,
- Insulin therapy and correct insulin administration techniques,
- Home blood glucose monitoring,
- Target blood glucose values,
- Recognition and management of acute diabetes-related emergencies,
- Prevention of chronic complications,
- The role of nutrition and physical activity in diabetes management,
- Foot care and long-term self-care behaviors.

**Educational Objectives**

The game aimed to enable participants to:

- Explain basic information about diabetes,
- Identify diabetes-related emergencies and appropriate interventions,
- Demonstrate correct steps for home blood glucose measurement,
- Recognize target blood glucose levels,
- Correctly explain and demonstrate insulin administration techniques,
- Understand the importance of regular exercise,
- Recognize long-term complications of diabetes,
- Apply appropriate foot care practices.

**Educational Approach**

The game integrates key elements of game-based learning, including competition, teamwork, repetition, and immediate feedback. These mechanisms were intended to enhance motivation, support knowledge retention, and facilitate behavioral engagement during the learning process. The intervention was delivered by trained nurse educators within diabetes education sessions.

**Description of the Control Group Educational Program**

Participants assigned to the control group received standardized diabetes education delivered through traditional lecture-based presentations. The educational content was designed to reflect routine diabetes education practices and was matched in duration and frequency to the intervention group.

**Structure of the Educational Sessions**

The control group participated in four weekly educational sessions, each lasting approximately two hours. The sessions were delivered by trained healthcare professionals and included didactic presentations and structured question-and-answer periods.

**Educational Content by Session**

- **Week 1:** General information about diabetes mellitus, disease mechanisms, and basic self-care principles.
- **Week 2:** Medical treatment of diabetes, with a focus on insulin therapy and insulin administration techniques.
- **Week 3:** Acute diabetes-related emergencies, chronic complications, preventive measures, and risk reduction strategies.
- **Week 4:** Exercise and physical activity recommendations, medical nutrition therapy, home blood glucose monitoring, overall review of educational content, and an interactive question-and-answer session.

**Assessment**

Post-intervention assessments, including the Patient Identification Form, Beck Anxiety Scale, and Patient Compliance Scale for Type 2 Diabetes Mellitus Treatment, were administered approximately one month after completion of the educational sessions.
