# Supplementary material for: The effect of game-based education on adherence to treatment and anxiety level in type 2 diabetics started on insulin therapy
Source: PLoS One. 2026 Mar 30;21(3):e0345292. doi: 10.1371/journal.pone.0345292 (PMC13035163; doi:10.1371/journal.pone.0345292)
Supplement: S3 File — (PDF) [file pone.0345292.s003.pdf]

REVİZYON MEKTUBU

- *Dışlanma kriterleri eklenmiştir.*
- *Eğitim programının içeriği ve nasıl uygulanacağı ayrıntılı bir şekilde tablo ve açıklama olarak anlatılmıştır.*
- *Eğitim seansları, deney grubunda oyun seviyelerine göre, kontrol grubunda anlatılan konuya göre haftalık programla açıklanmıştır.*
- *Toplamda yedi görüşme ön test- son test değerlendirmesi (başlangıç ve en sonda iki görüşme), beş oturumda eğitimlerin deney ve kontrol gruplarına yönelik açıklaması yapılmıştır.*
- *Deney grubunda uygulanacak olan Diyabeti Öğrenelim Masa oyunu hakkında ayrıntılı bilgi verilmiştir.*
- *Araştırmanın kapsamı kısmında diğer maddesi altında eklenen bilgiler silinmiştir.*
- *Tez sürecinde iş-zaman plan çizelgesi tablo ile açıklanmıştır.*

Sorumlu Araştırmacı:

Yardımcı Araştırmacı:

DİYABETİ ÖĞRENELİM OYUNU

(DENEY GRUBUNA EĞİTİMDE UYGULANACAK OYUN TEMELLİ TASARIM)

SÜRE: 45-60 DAKİKA

AMAÇ: Eğitim sürecinin motive edici hala gelmesini sağlayan yöntemlerden biri de eğitimde oyunlaştırmanın kullanılmaya başlanması olmuştur. Eğitimde oyunlaştırma ile amaç bireyi eğitim sürecinde daha aktif hale getirmek ve eğitimi stres verici bir durum olmaktan çıkartmaktır. Ayrıca bu yöntemle bireyin eğlenirken öğrenmesi, eğitimde verimin artırılması hedeflenmektedir. Yeni bir bilgi öğrenmek güçlü bir fiziksel, bilişsel çaba ve özdenetim gerektirmektedir. Oyunun amacı Diyabetli bireyler, öğretmenler, öğrenciler, oyunu oynayan ve oynatan tüm kişiler için diyabette farkındalık oluşturmak. Diyabetli bireyler için diyabetle yaşamı kolaylaştıracak temel bilgilerin interaktif bir biçimde oyunlaştırılmasını sağlamak. Ayrıca diyabet okullarında diyabet hemşireleri tarafından verilen eğitimlerin diyabetli bireyleri oyun içine katarak motivasyonlarını sağlamak.

MALZEMELER: Yer ya da masa üzerinde kullanılabilecek bir ana zemin oyun halısı, zar, her bir seviye için 23 adet soru cevap kartları toplamda 69 kart (birinci seviye kolay, ikinci seviye orta, üçüncü seviye zor kategoride soru ve konuları içerir), flipchart yazı tahtası

AÇIKLAMA: Oyuncular katılımcı sayısına göre iki ya da üç takıma ayrılır. Takımlardan birer sözcü seçilir ve zar atılır. Oyuna başlayacak takım zarla seçildikten sonra oyun halısının üzerinde 1'den 23'e kadar olan rakamlardan sırasıyla her takım ortak karar vererek birini seçip o rakamın yazılı olduğu kartları açtırır. Kartlarda yer alan soruyu doğru yanıtlayan ve yönergeyi yapan takım puan kazanır. Sırayla rakamlar açtırılır ve bitene kadar takımların puanları kolaylaştırıcı tarafından kayıt edilir.

KAZANIMLAR:

- Diyabetle ilgili temel bilgileri açıklar
- Diyabette acil durumları ve müdahalelerini açıklar
- Evde kan şekeri ölçümüyle ilgili doğru basamakları açıklar ve uygular
- Diyabetli bireylerde hedef kan şekeri değerlerini açıklar.
- İnsülin uygulama basamaklarını anlatır ve gösterir.
- Diyabette egzersizin önemini bilir ve anlatır.
- Diyabetin sebep olduğu uzun vadeli sağlık sorunlarının önemini bilir

- Diyabette ayak bakımını anlatır ve uygular.

Sorumlu Araştırmacı:

Yardımcı Araştırmacı:

Tez SÜl'ecindeki İşlerin İş-Zaman Plan Çizelgesi

İş Paketi No	İş paketi adı, tanımı	Ocak-Mart 2024	Şubat-I\,I;ırt 2024	Mayıs-Temmuz 2024	Temmuz 2024	Ağustos-Eylül-Ekim 2024	Ekim-K..-ı.sım	Aralık 2025	Ocak-Subat ·2025
1	Lltentür tarama	✓							
2	Kaynakları tarama ve not alına			✓					
3	Tez konusunun netle.ştirilmesi	✓							
4	Evren belirleme ve ömeklem seçimi			✓					
5	Etik k:ıırul ve kurum izinlerinin alınması		✓						
6	Deney ve Kontrol gruplarına ön test ve ölçeklerin uygulanması				✓				
7	Deney ve kontrol gruplarına beş oturumda eğitimlerin uygulanması, son test ölçeklerin uygulanması					✓			
8	Veri girişi ve analizi						✓		
9	Tezin yazılması							✓	
9	Tezin İZTÜ Lisansüstü Eğitim Enstitüsüne'ne teslimi								✓

Şekil:2

AÇIKLAMA: Oyuncular katılımcı sayısına göre iki ya da üç takıma ayrılır. Takımlardan birer sözcü seçilir ve zar atılır. Oyuna başlayacak takım zarla seçildikten sonra oyun halısının üzerinde 1'den 23'e kadar olan rakamlardan sırasıyla her takım ortak karar vererek birini seçip o rakamın yazılı olduğu kartları açtırır. Kartlarda yer alan soruyu doğru yanıtlayan ve yönergeyi yapan takım puan kazanır. Sırayla rakamlar açtırılır ve bitene kadar takımların puanları kolaylaştırıcı tarafından kayıt edilir.

KAZANIMLAR:

- **Diyabetle** ilgili **temel bilgileri açıklar**
- **Diyabette acil durumları ve müdahalelerini açıklar**
- **Evde kan şekeri ölçümüyle ilgili doğru basamakları açıklar ve uygular**
- **Diyabetli bireylerde hedef kan şekeri değerlerini açıklar.**
- **İnsülin uygulama basamaklarını anlatır ve gösterir.**
- **Diyabette egzersizin önemini bilir ve anlatır.**
- **Diyabetin sebep olduğu uzun vadeli sağlık sorunlarının önemini bilir**
- **Diyabette ayak bakımını anlatır ve uygular.**

Araştırmada elde edilen veriler SPSS (Statistical Package for Social Sciences) for Windows 25.0 programı kullanılarak analiz edilecektir. Verileri değerlendirilirken tanımlayıcı istatistiksel metotları (sayı, yüzde, min-maks değerleri, ortalama ve standart sapma) kullanılacaktır. Ölçeklerin güvenilirliğini test etmek amacıyla "Güvenilirlik Analizi" uygulanacaktır.

- **Eylül ikinci hafta oyunun seviyelerinin karma bir şekilde uygulanması**
- **Ekim son haftasında son test olarak hasta tanılama formu, beck anksiyete ölçeği ve tip 2 diyabet tedavisinde hasta uyum ölçeğininin uygulanması**

Kontrol Grubu

- **Ağustos birinci hafta sunum yoluyla Diyabette genel bilgiler verilecektir**
- **Ağustos ikinci hafta diyabette ilaç tedavisi, insulin uygulamaları ile ilgili sunumun uygulanması**
- **Ağustos üçüncü hafta Diyabette acil durum ve müdahaleleri, kronik komplikasyonlar ve önlemleriyle ilgili sunumun uygulanması**
- **Ağustos dördüncü hafta egzersiz, tıbbi beslenme tedavisi ve evde kan şekeri ölçümüyle ilgili sunumun uygulanması**
- **Eylül ikinci hafta eğitimlerin sunum yoluyla genel tekrarı, soru ve cevapların uygulanması**
- **Ekim son haftasında son test olarak hasta tanılama formu, beck anksiyete ölçeği ve tip 2 diyabet tedavisinde hasta uyum ölçeğininin uygulanması**

DİYABETİ ÖĞRENELİM OYUNU

SÜRE: 45-60 DAKİKA

AMAÇ: Eğitim sürecinin motive edici hala gelmesini sağlayan yöntemlerden biri de eğitimde oyunlaştırmanın kullanılmaya başlanması olmuştur. Eğitimde oyunlaştırma ile amaç bireyi eğitim sürecinde daha aktif hale getirmek ve eğitimi stres verici bir durum olmaktan çıkartmaktır. Ayrıca bu yöntemle bireyin eğlenirken öğrenmesi, eğitimde verimin artırılması hedeflenmektedir. Yeni bir bilgi öğrenmek güçlü bir fiziksel, bilişsel çaba ve özdenetim gerektirmektedir. Oyunun amacı Diyabetli bireyler, öğretmenler, öğrenciler, oyunu oynayan ve oynatan tüm kişiler için diyabette farkındalık oluşturmak. Diyabetli bireyler için diyabetle yaşamı kolaylaştıracak temel bilgilerin interaktif bir biçimde oyunlaştırılmasını sağlamak. Ayrıca diyabet okullarında diyabet hemşireleri tarafından verilen eğitimlerin diyabetli bireyleri oyun içine katarak motivasyonlarını sağlamak.

MALZEMELER: Yer ya da masa üzerinde kullanılabilecek bir ana zemin oyun halısı, zar, her bir seviye için 23 adet soru cevap kartları toplamda 69 kart (birinci seviye kolay, ikinci seviye orta, üçüncü seviye zor kategoride soru ve konuları içerir), flipchart yazı tahtası

konusunda olumlu yönde etkisi vardır.

H0: Tip 2 diyabetli bireylere verilen oyun tabanlı eğitimin, bireylerin anksiyete düzeyine olumlu yönde etkisi yoktur.

Hl: Tip 2 diyabetli bireylere verilen oyun tabanlı eğitimin, bireylerin anksiyete düzeyine olumlu yönde etkisi vardır

Uşak Eğitim ve Araştırma Hastanesi Endokrin ve Dahiliye poliklinik/kliniklerine başvurup son üç ayda insülin tedavisine başlanan ve Diyabet Eğitim Birimine başvuran Tip 2 Diyabetli hastalar içinden,

Dahil Edilme Kriterleri

- Diyabet tanı yaşının en az bir yıl olması
- İnsülin tedavisi başlanma süresi an fazla üç ay olması
- Mental herhangi bir sorununun olmaması
- 40- 65 yaş aralığında olması
- Araştırmaya katılmaya gönüllü olması gibi kriterleri karşılayan hastalar araştırmanın kapsamına alınacaktır.

Dışlanma Kriterleri

- **Diyabet tanı yaşının yeni veya bir yıldan daha az süre olması**
- **Üç aydan daha uzun süredir insülin tedavisi alıyor olması**
- **Mental yada psikolojik yönden ek hastalığının olması**
- **Hedef yaş aralığında olmaması, 40 yaş altı yada 65 yaş üzerinde olması**
- **Araştırmaya katılmayı kabul etmemek**
- **Araştırma sürecinde beş oturumdan oluşan eğitim seanslarından biri veya daha fazlasına katılmamak**

Veri Toplama Araç ve Yöntemleri - Kullanılacak İstatiksel Yöntemler

Ön test- Son Test Randomize Kontrol grupıu Deneysel Çalışmamızda kullanılacak form ve ölçekler Gönüllü Onam Formu, Hasta Tanılama Formu, Beck Anksiyete Ölçeği, Tip 2 Diabetes Mellitus Tedavisine Hasta Uyum Ölçeğidir.

unsurları kişilerin motivasyonunu arttırmaktadır (4,5). Yapılan literatür taramalarında oyunlaştırma uygulamalarının diyabetten korunma amaçlı sağlıklı beslenme, fiziksel aktivite programlarıyla birlikte geliştirilerek hayata geçirilebileceği ifade edilebilir. Hastaların beslenme planları, kan şekeri ölçümleri ve kullandıkları insülin miktarlarının kaydedildiği oyunlaştıma uygulamaları, diyabetle mücadelede bireylerin motivasyonunu artırarak tedavi etkinliğini artırabilir, hasta takibini kolaylaştırabilir ve gereksiz hastane ziyaretlerini azaltabilir. Bu, sağlık hizmetlerinin etkinliğine ve verimliliğine katkıda bulunabilir. Burada dikkat edilmesi gereken konular arasında ise hedef, eğlence ve uygun araçları içerecek şekilde doğru bir oyunlaştırma süreç ve tasarımının kurulması yer almaktadır. Bununla birlikte, diyabet hastalığı bağlamında oyunlaştırma uygulamasına giriş sıklığının yüksekliği nedeniyle karmaşık olmayan, doğru tutum ve davranışların ödüllendirildiği uygulamaların hayata geçirilmesinin bu oyunlaştırmaların etkinliğini arttıracağı ifade edilmektedir (6). Oyun Tabanlı Eğitim Modelleri eğitim alanında farklı alanlarda çok sık kullanılırken sağlık alanında çok sınırlı sayıdadır. Oysaki Diyabet gibi yönetimi zor olan bir hastalıkta uyum ve anksiyete düzeyi çok önemlidir. Tüm diyabetliler özellikle yaş, fiziksel engel, komplikasyonlar nedeniyle teknolojiye hakim olamayabilirler. Eğlenerek öğrenmek onların da hakkı olduğu için oyunlar her yaştan, her kültürden diyabetli bireye uygun tasarlanmış, basit ve anlaşılır bir yapıya sahiptir. Ancak dijital ortam dışında masa yada kutu oyununa rastlanmamıştır. Bu nedenlerle araştırmacı ve arkadaşları tarafından tasarlanan diyabetle ilgili oyunların Tip 2 diyabetli bireylerin tedaviye uyumları, anksiyete düzeylerine etkilerinin incelenmesi amacıyla randomize kontrollü deneysel bir çalışma yapılacaktır. Oyun Tabanlı Eğitim modellerinin tip 2 diyabetli bireyin tedaviye uyumunu olumlu yönde etkileyeceği ve anksiyete düzeyini azaltma konusunda ilave katkı sağlayacağı düşünülmektedir.

ARAŞTIRMANIN GEREÇ VE YÖNTEMİ

Hipotezler:

H0: Tip 2 diyabetli bireylere verilen oyun tabanlı eğitimin, bireylerin diyabet tedavisine uyumu konusunda olumlu yönde etkisi yoktur.

Hl: Tip 2 diyabetli bireylere verilen oyun tabanlı eğitimin, bireylerin diyabet tedavisine uyumu

araştırma

O Anket yöntemi

O Diğer:

ARAŞTIRMANIN AMACI/ GEREKÇESİ:

Bu çalışmada insülin tedavisi başlanan tip 2 diyabetlilerde oyw1 tabanlı eğitimin tedaviye uyuma ve anksiyete düzeyine etkisini incelemek amaçlanmaktadır . Araştım1amız Uşak Eğitim Araştırma Hastanesi Endokrin ve Dahiliye Polikliniklerine başvuran en fazla üç ay öncesinde ilk kez insülin tedavisi başlanan tip 2 diyabetlileri kapsayacak şekilde deneysel bir çalışma olarak yürütülecektir.

Günümüzün en önemli sağlık sornnlarından biri olan diyabet, Dünya Sağlık Örgüt'üne göre "Bulaşıcı olmayan, salgın hastalık" olarak tanımlanmıştır (1). Uluslararası Diyabet Federasyonu (Intemational Diabetes Federation-IDF) verilerine göre, 2021 yılında yaklaşık 537 milyon kişinin diyabet olduğu tahmin edilmektedir. Bu sayının 2030 yılına kadar 643 milyona ve 2045 yılına kadar da 783 milyona ulaşması beklenmektedir. Bu veriler, diyabetin küresel anlamda artan bir sağlık sornnu olduğunu ve önlem alınması gerektiğini göstermektedir. IDF Diyabet Atlası (2021), yetişkin nüfusun (20-79 yaş) %10,5' inin diyabet olduğunu ve neredeyse yarısının bu hastalıkla yaşadıklarının farkında olmadığını bildirmektedir (2)

Diyabetlilerin, yaşam kalitesinin yükseltilmesi, komplikasyonlardan korunması ve metabolik kontrolün sağlanması için etkili ve sürekli bir bakıma ve izlem programına ihtiyaçları vardır. Bu izlem programında diyabet eğitiminin önemi kaçınılmazdır. Eğitim, diyabetli birey ve ailesi için bakım ve izlemi kolaylaştıracak, devamlılığını sağlayacak bilgi, tutum ve becerilerin tümünü kapsamalıdır (3). Eğitsel oyunların hakim olduğu öğrenme ortan1larına oyun-tabanlı öğrenme ortamları denir. Oyun-tabanlı eğitim modelinde öğrenen aktif durumdadır. Oyun esnasında geribildirim aldıkları için hatalarını düzeltme olanakları vardır. Ayrıca yarış, rekabet gibi oyun

ARAŞTIRMANIN NİTELİĞİ

☐ Münferit Araştırma

☐ Doktora Tezi

☐ Uzmanlık Tezi

■ Yüksek Lisans Tezi

☐ Diğer (Türünü belirtiniz):

ARAŞTIRMANIN ÖNGÖRÜLEN SÜRESİ:

Planlanan Başlangıç Tarih:

Planlanan Bitiş Tarihi:

Bütçesi ve Kaynağı: Bütçe ve desteği bulunmamaktadır.

ARAŞTIRMANIN KAPSAMI

☐ Gözlemsel araştırma

☐ Dosya ve görüntü kayıtları gibi retrospektif arşiv taraması

☐ Kan, idrar, doku, görüntü gibi biyokimya, mikrobiyoloji, patoloji ve radyoloji koleksiyon materyalleriyle veya rutin muayene, tetkik, tahlil ve tedavi işlemleri sırasında elde edilmiş mateıyalle yapılacak araştırma

☐ Hücre veya doku kültürü araştırması

☐ Gen tedavisi klinik araştırmaları dışında kalan ve tanımlamaya yönelik genetik mateıyalle yapılacak araştırma

☐ Hemşirelik faaliyetlerinin sınırları içerisinde yapılacak araştırma

☐ Bilgisayar ortamında test, mülakat, ses veya görüntü kaydı ile toplanacak verilerin kullanılacağı araştırma

☐ Biyomedikal ve/veya ekoloji ile ilgili araştırma

☐ Sağlık eğitimi faaliyetleri ile ilgili araştırma

☐ Antropometrik ölçümlere dayalı araştırma

☐ Egzersiz gibi vücut fizyolojisi ile ilgili araştırma

☐ Beslenme/diyet ile ilgili araştırma

☐ Yaşanı alışkanlıklarının değerlendirilmesi ile ilgili araştım1a

☐ Vücut dışında kullanılan tıbbi tanı cihazları ile yapılan perfom1ans değerlendirmesi ile ilgili

Diğer Araştırmacılar:			
Adı Soyadı:			
Unvanı:			
Doktora/Uzmanlık/Diğer Alanı:			
İş adresi:			
Telefon numarası:			
E-posta:			
Araştırmadaki Sorumluluğu/Katkısı:			
☐	Hipotez geliştirme	☐	Araştırmayı planlama
☐	Veri toplama	☐	Veri analizi ve yorumlama
☐	Makale yazımı	■	Diğer: Yüksek Lisans Tezi
İmza:			

**Başvuru Tarihi**
Revizyon Tarihi

**Araştırmanın açık adı:** İnsülin Tedavisi Başlanan Tip 2 Diyabetlilerde Oyun Tabanlı Eğitimin Tedaviye Uyuma ve Anksiyete Düzeyine Etkisi

**Sorumlu Araştırmacı**			
Adı, Soyadı:			
Unvanı:			
Doktora/Uzmanlık Alanı: :			
İş adresi			
Telefon numarası:			
E-posta:			
Araştırmadaki Sorumluluğu/Katkısı:			
☐	Hipotez geliştirme	☐	Araştırmayı planlama
☐	Veri toplama	☐	Veri analizi ve yorumlama
☐	Makale yazımı	☐	Diğer: Yüksek Lisans Tez Danışmanı
İmza:			

Şekil: t

1 Eğitim Programı içeriği ve İşlem Basamakları

\ Hastalar Deney ve Kontrol Grubu olmak üzere iki gruba ayrılacak, tümüne ön test olarak form ve ölçekler çalışma öncesi doldurulacak (Temmuz son hafta).

Dene) grubuna araştırmacı tarafından tasarlanan Diyabeti Öğrenelim Masa 0) unuyla birinci ay dört oturum, ikinci ay bir oturum olmak üzere toplamda beş oturum eğitim Uygulanacaktır. Kontrol grubuna ise aynı sürede sunum (anlatım) yoluyla eğitim uygulanacaktır (Şekil: 1). Oturum süreleri ortalama iki saat olarak planlanmıştır. Üçüncü ayda son test olarak form ve ölçeklerin son değerlendirmeleri yapılacaktır. Dene) ve Kontrol gruplarına bu eğitimler Uşak Eğitim ve Araştırma Hastanesi Diyabet Okulu salonunda Uygulanacaktır. Diyabet Okulu salonu toplamda 16 kişilik olduğu için power analizi sonucu örneklem sayısının 64 (32 Deney grubu, 32 Kontrol grubu) olması ön görülerek her bir oturum deney grubu için sabah ve öğleden sonra (16-16), kontrol grubu için sabah \'e öğleden sonra (16-16) olmak üzere iki gruba ayrılarak deney grubu farklı bir günde kontrol grubu farklı bir günde olacak şekilde ilk ay haftada birer gün, ikinci ay a)da birer gün her bir grup toplamda beş oturum eğitime katılacaklardır.

Deney Grubu

- Ağustos birinci hafta oyunun tanıtılması ve birinci seviyenin uygulanması
- Ağustos ikinci hafta oyunun ikinci seviyesinin uygulanması
- Ağustos üçüncü hafta oyunun üçüncü seviyesinin uygulanması
- Ağustos son hafta oyunun seviyelerinin karma bir şekilde uygulanması
