# Supplementary material for: The effect of game-based education on adherence to treatment and anxiety level in type 2 diabetics started on insulin therapy
Source: PLoS One. 2026 Mar 30;21(3):e0345292. doi: 10.1371/journal.pone.0345292 (PMC13035163; doi:10.1371/journal.pone.0345292)
Supplement: Attachment — Submitted filename: renponses.docx [file pone.0345292.s004.docx]

**Response to Reviewers**

Manuscript Title:
**The effect of game-based education on adherence to treatment and anxiety level in type 2 diabetics started on insulin therapy**

Dear Academic Editor and Reviewers,

We would like to thank the Academic Editor and both reviewers for their careful evaluation of our manuscript and for their constructive and insightful comments. We have revised the manuscript thoroughly in response to these suggestions. Below, we address each comment point-by-point and describe the corresponding revisions made. All changes are highlighted in the revised manuscript with track changes.

**Responses to Reviewer 1**

We thank Reviewer 1 for the detailed methodological and statistical critique, which substantially strengthened the rigor and clarity of our manuscript.

**Comment 1: Prespecification of a single primary objective and endpoint; control of multiplicity**

**Response:**
We agree with this important point. The manuscript has been revised to clearly prespecify:

- **Primary objective:** To evaluate the effect of game-based education on treatment adherence in adults with type 2 diabetes initiating insulin therapy.
- **Primary endpoint:** Post-intervention total score of the Patient Compliance Scale for Type 2 Diabetes Treatment.

The sample size calculation was aligned with this primary endpoint and explicitly described as such. All other outcomes, including anxiety and compliance subscales, are now clearly labeled as **secondary or exploratory outcomes**.

Regarding multiplicity, we acknowledge that no formal familywise error correction was applied. In line with the reviewer’s recommendation, exploratory analyses of multiple subscales are explicitly identified as hypothesis-generating, and p-values are interpreted cautiously. This clarification has been added to the **Statistical Analysis** and **Results** sections.

**Location of revision:** Methods (Objectives, Statistical Analysis), Results (Exploratory Analyses).

**Comment 2: Use of baseline-adjusted ANCOVA instead of multiple t-tests**

**Response:**
We fully agree. The analytical approach has been revised accordingly. The primary between-group analysis is now conducted using **analysis of covariance (ANCOVA)** on post-test outcomes, adjusting for baseline values and prespecified covariates (age, sex, duration of diabetes). Employment status, which differed at baseline, was additionally included as a covariate.

Adjusted mean differences with 95% confidence intervals and effect size estimates (partial η²) are now reported. The Results and Tables have been revised to reflect this approach, and multiple unadjusted t-tests have been removed from inferential interpretation.

**Location of revision:** Statistical Analysis; Results; Tables 3 and 4.

**Comment 3: Randomization details and CONSORT flow diagram**

**Response:**
Additional details regarding randomization have been added, including sequence generation, allocation ratio, concealment, and personnel roles. A CONSORT-style participant flow description has been incorporated into the Methods section, detailing screening, randomization, exclusions, and final analysis numbers. A flow diagram has been prepared and included as Supporting Information.

**Location of revision:** Methods (Randomization and Allocation); Supporting Information.

**Comment 4: Power calculation alignment with primary endpoint**

**Response:**
The sample size calculation has been revised to explicitly align with the prespecified primary endpoint and ANCOVA framework. Assumptions regarding effect size, alpha level, power, and attrition have been clarified. No interim analyses were planned or conducted, and this has now been stated explicitly.

**Location of revision:** Methods (Sample Size Calculation).

**Comment 5: Analysis population and missing data**

**Response:**
We have clarified that analyses were conducted on an **intention-to-treat basis**. As no post-randomization attrition occurred, no imputation procedures were required. This has been explicitly stated to improve transparency.

**Location of revision:** Statistical Analysis.

**Comment 6: Prespecified covariates and multivariable analysis**

**Response:**
A limited and clinically justified set of covariates (age, sex, diabetes duration, and employment status) was prespecified and consistently applied across ANCOVA models. No post hoc variable selection was performed. This clarification has been added to the Statistical Analysis section.

**Comment 7: Emphasis on estimates rather than p-values**

**Response:**
We revised the Results and Discussion sections to emphasize **adjusted effect estimates, confidence intervals, and clinical interpretation**, rather than statistical significance alone. Effect sizes are discussed in terms of clinical relevance where appropriate.

**Responses to Reviewer 2**

We thank Reviewer 2 for their positive evaluation and constructive conceptual suggestions.

**Comment: Clarification that reference [12] is a PhD thesis**

**Response:**
This has been corrected. Reference [12] is now clearly identified as a doctoral thesis.

**Comment: More detail on game mechanisms and materials**

**Response:**
Additional description of the game mechanics (competition, teamwork, feedback, progression) has been added to the Intervention Procedures section. Representative images of the board game have been included as Supporting Information.

**Comment: Conceptual clarity regarding adherence vs compliance**

**Response:**
We appreciate this important conceptual clarification. The Introduction and Discussion sections were revised to explicitly distinguish between treatment adherence, patient compliance, and broader constructs such as patient engagement. We clarify that the present study operationalized adherence primarily in behavioral terms using the Patient Compliance Scale, while cognitive and emotional components were not directly measured and represent directions for future research.

**Comment: Results readability and separation from Discussion**

**Response:**
The Results section has been rewritten to improve readability and reduce repetition. Interpretative statements and explanations have been moved to the Discussion section, ensuring a clear structural separation between results and interpretation.

**Comment: Limitations and implications**

**Response:**
A comprehensive **Limitations** section has been added, and the **Implications for Future Research** section has been expanded to reflect theoretical and clinical relevance.

**Data Availability and Journal Requirements**

We revised the Data Availability Statement to comply with PLOS ONE policy. The institutional ethics committee is now listed as the non-author contact point for data access. All tables have been embedded in the main manuscript, and the study protocol has been uploaded as Supporting Information.

We sincerely thank the reviewers and the Academic Editor for their valuable feedback, which has substantially improved the quality and clarity of our manuscript. We hope that the revised version adequately addresses all concerns and we look forward to further consideration.

Kind regards,
