# Supplementary material for: The effect of game-based education on adherence to treatment and anxiety level in type 2 diabetics started on insulin therapy
Source: PLoS One. 2026 Mar 30;21(3):e0345292. doi: 10.1371/journal.pone.0345292 (PMC13035163; doi:10.1371/journal.pone.0345292)
Supplement: Attachment — Submitted filename: response to reviwers.docx [file pone.0345292.s005.docx]

**Manuscript ID:** PONE-D-25-52606R1
**Title:** The effect of game-based education on adherence to treatment and anxiety level in type 2 diabetics started on insulin therapy

Dear Academic Editor and Reviewer,

We sincerely thank the Academic Editor and Reviewer for their careful re-evaluation of our manuscript and for their constructive feedback. Below, we provide a point-by-point response to each comment. All revisions have been incorporated into the manuscript and highlighted in the tracked-changes version.

**Reviewer Comment 1**

The authors should ensure the manuscript consistently treats the total Patient Compliance Scale as the single primary confirmatory outcome, and clearly label all other outcomes and subscales as secondary or exploratory across the Abstract, Results, and Discussion.

**Response:**

We agree with this recommendation. The total score of the Patient Compliance Scale for Type 2 Diabetes Mellitus Treatment is now consistently defined as the sole primary confirmatory outcome throughout the manuscript. Anxiety outcomes and all compliance subscale analyses are explicitly described as secondary or exploratory in the Abstract, Results, and Discussion sections. *(Revised in: Abstract; Results; Discussion)*

**Reviewer Comment 2**

The authors should align the results tables with the stated analytic strategy by removing remaining unadjusted post-intervention t-test inferences from the main tables, or explicitly labeling them as descriptive only and basing conclusions on the ANCOVA results.

**Response:**

In line with this suggestion, all unadjusted post-intervention t-test inferences have been removed from the main results tables. Between-group comparisons and statistical inferences are now exclusively based on baseline-adjusted ANCOVA models. The analytic approach is clearly described in the Results section and explicitly stated in the relevant table footnotes. *(Revised in: Results; Table 3; Table 4)*

**Reviewer Comment 3**

The authors should rewrite the sample size justification to match the specified primary endpoint and analysis, including what outcome the assumed effect size pertains to, how it was derived, and whether the intended powering framework was ANCOVA with baseline adjustment or a two-sample t-test approximation.

**Response:**

We have revised the sample size justification to clearly align with the primary endpoint, defined as the total score of the Patient Compliance Scale. The assumed effect size (Cohen’s d = 0.70) is now explicitly linked to this outcome and described as being informed by prior behavioral intervention studies in diabetes education using a conservative two-sample t-test approximation. We also clarify that, although the primary analysis employed baseline-adjusted ANCOVA, the t-test–based sample size estimation was considered appropriate and conservative. *(Revised in: Methods – Sample Size subsection)*

**Reviewer Comment 4**

The authors should resolve the covariate mismatch between the Statistical Analysis section and table footnotes by making them identical, and confirm the exact covariate set used to generate the reported adjusted mean differences and p values.

**Response:**

This issue has been fully addressed. The Statistical Analysis section and all table footnotes have been harmonized to report an identical set of covariates used in baseline-adjusted ANCOVA models. These covariates are baseline outcome score, age, duration of diabetes, and employment status, the latter of which differed significantly between groups at baseline. *(Revised in: Methods – Statistical Analysis; Table 3; Table 4)*

**Reviewer Comment 5**

The authors should report a consistent effect size metric for the primary outcome analysis and ensure any effect size claims in the text match what is shown in the tables.

**Response:**

We have ensured consistent reporting of effect size metrics across the manuscript. For the primary outcome, interpretation is based on adjusted ANCOVA results, and all effect size claims in the Results and Discussion sections directly correspond to the metrics presented in the tables. For anxiety outcomes, partial η² values are consistently reported and interpreted in both the text and tables. *(Revised in: Results; Discussion; Table 3; Table 4)*

Once again, we thank the Academic Editor and Reviewer for their valuable comments. We believe these revisions have substantially improved the clarity, consistency, and interpretability of the manuscript, and we hope that the revised version is now suitable for publication in *PLOS ONE*.

Sincerely,
